# Expression of *Piwi* Genes during the Regeneration of *Lineus sanguineus* (Nemertea, Pilidiophora, Heteronemertea)

**DOI:** 10.3390/genes11121484

**Published:** 2020-12-10

**Authors:** Cong-Mei Xu, Shi-Chun Sun

**Affiliations:** College of Fisheries, Institute of Evolution and Marine Biodiversity, Ocean University of China, 5 Yushan Road, Qingdao 266003, China; vickey1202@126.com

**Keywords:** Nemertea, *Lineus sanguineus*, *piwi*, regeneration, stem cell

## Abstract

The transposon silencer *piwi* genes play important roles in germline determination and maintenance, gametogenesis, and stem-cell self-renewal, and the expression of certain *piwi* genes is indispensable for regeneration. Knowledge about *piwi* genes is needed for phylum Nemertea, which contains members (e.g., *Lineus sanguineus*) with formidable regeneration capacity. By searching the *L. sanguineus* genome, we identified six Argonaute genes including three *ago* (*Ls-Ago2*, *Ls-Ago2a*, and *Ls-Ago2b*) and three *piwi* (*Ls-piwi1*, *Ls-piwi2*, and *Ls-piwi3*) genes. In situ hybridization revealed that, in intact females, *Ls-piwi2* and *Ls-piwi3* were not expressed, while *Ls-piwi1* was expressed in ovaries. During regeneration, *Ls-piwi1* and *Ls-pcna* (proliferating cell nuclear antigen) had strong and similar expressions. The expression of *Ls-piwi1* became indetectable while *Ls-pcna* continued to be expressed when the differentiation of new organs was finished. During anterior regeneration, expression signals of *Ls-piwi2* and *Ls-piwi3* were weak and only detected in the blastema stage. During posterior regeneration, no expression was observed for *Ls-piwi2*. To date, no direct evidence has been found for the existence of congenital stem cells in adult *L. sanguineus*. The “pluripotent cells” in regenerating tissues are likely to be dedifferentiated from other type(s) of cells.

## 1. Introduction

The ribbon worm *Lineus sanguineus* (Rathke, 1799) (Nemertea, Pilidiophora, Heteronemertea) is famous for its formidable regenerative ability. This species is able to regenerate an entire individual not only from a thin transverse slice, but even from just one quadrant of a thin slice as long as the regenerating part includes a part of the cerebral ganglia or lateral nerve cords [1]. By repeatedly amputating a single worm, over 200,000 worms could be obtained, each measuring less than one two-hundred-thousandth of the volume of the original individual [2]. This nemertean reproduces asexually via spontaneous autotomy (fragmentation) in its natural life cycle [3].

Upon experiencing an injury of amputation, worms close the wound by contraction of the body wall musculature that brings together epidermal structures, causing compression of the underlying tissues, which seals off the cavities of the blood vessels, gut, and rhynchocoel [2]. Wound healing is accomplished by cellular migration and involves cells originating from both the parenchyma and the proximal regions of the epidermis [1]. Undifferentiated epidermal cells, termed neoblasts, are primarily responsible for the development of the thin flattened epithelium that quickly forms over the wound [1]. In regenerating anterior structures, undifferentiated migratory cells beneath the newly formed epidermis give rise to a projecting regenerative bud or head blastema, which eventually gives rise to all the structures of the new anterior end [2,4,5]. Beyond this general description, very little is known about the cellular underpinnings of regeneration in nemerteans. According to histological studies, some authors proposed that cells scattered within the relatively abundant extracellular matrix are pluripotent, contributing to normal growth and migrating to wound sites to give rise to regenerated tissues [5]. This model would be very similar to the neoblast model in planarians [6]. However, a more recent study on the morphology of the cells within the extracellular matrix did not support that they were undifferentiated stem cells [7]. To gain a better understanding of the mechanisms of nemertean regeneration, molecular studies are needed.

Members of the Argonaute protein family play a crucial role during development, stem-cell maintenance, and differentiation, as well as for endogenous RNA interference and mobilization control of retrotransposons in many organisms [8]. The Argonaute protein family is further classified into two subfamilies, the Ago subfamily and the Piwi subfamily, on the basis of several conserved domains [9]. The Ago subfamily is evolutionarily conserved within the eukaryotes and expressed ubiquitously in many different tissues, and its members bind to microRNA (miRNA)/small interfering RNA (siRNA) to guide post-transcriptional gene silencing either via restabilization of the mRNA or via translational repression [10]. In contrast, the Piwi subfamily members, originally named after *Drosophila melanogaster* Piwi (P-element induced wimpy testis), bind to piRNAs (Piwi-interacting RNAs) [11] and are mostly restricted to germ cells and stem cells; they affect germline determination, germline maintenance, gametogenesis, and stem-cell self-renewal in most animals studied [12]. In adult *D. melanogaster*, *Piwi* is enriched in the nucleus of both the germ and the somatic cells of gonads [13]. The zebrafish *piwi* gene, *Ziwi*, is expressed in early embryos and in both female and male germ cells of the adults, with the highest expression seen during the mitotic and early meiotic stages of germ cell [14]. *Piwi* genes are also expressed in germ cells of other animals such as sea urchins [15], ctenophores [16], and jellyfish [17]. Moreover, *piwi* genes are the most famous markers of undifferentiated cells in invertebrates [11]. In the planarian *Schmidtea mediterranea*, two *piwi* homologs, *Smedwi-2* and *Smedwi-3*, are expressed in neoblasts and are required for neoblast function in regeneration; RNA interference (RNAi) of *Smedwi-2* may cause the failing of tissue homeostasis and the loss of complete regeneration capacity [18,19]. To date, the information about *piwi* genes is completely unknown in nemerteans.

In this study, we identified *piwi* genes from the *L. sanguineus* genome. Their expression patterns in both intact worms and regenerating body fragments were determined by whole-mount in situ hybridization. To gain a better understanding of the cell proliferation during regeneration, the *pcna* gene (homolog of proliferating cell nuclear antigen), which is a well-known marker of proliferating cells [20], was studied in parallel.

## 2. Materials and Methods

### 2.1. Experimental Animals

Worms of *Lineus sanguineus* were collected in Qingdao, China. Their asexual progenies were kept in laboratory conditions (salinity of 30, temperature of 19 °C) and fed with freshwater oligochaetes. The identification of species was confirmed by the barcoding COI (cytochrome c oxidase subunit I) sequence (GenBank accession number: MW193720), which shows >99% similarity with sequences (e.g., KR606053, KP213895, and KX261775) already proven to be *L. sanguineus* (=*Ramphogordius sanguineus*) [21,22].

### 2.2. Gene Identification Analysis

BLASTp searches of the *L. sanguineus* genomic database (authors’ unpublished data) were conducted to find homologs of *D. melanogaster* PIWI (NCBI accession number AF104355) and PCNA (NCBI accession number NM_057557). For the purpose of comparison, the genome of another nemertean, *Notospermus geniculatus* (Delle Chiaje, 1828) (https://marinegenomics.oist.jp/nge_v2/viewer/download?project_id=52), was also searched. Amino-acid sequences for related proteins across a broad diversity of animal taxa were downloaded from the protein database in GenBank (for accession numbers, see Table S1, Supplementary Materials). Each domain was identified by *Pfam* searches using default parameters. Amino-acid sequence alignment was carried out using MAFFT v. 7 [23] followed by Gbloks v. 0.91b [24] to gain the conserved sequences of the protein set. ModelFinder [25] was used to determine the appropriate model of protein evolution, and the RtRev + G model was recommended and used for Bayesian analyses. Bayesian analysis was conducted with MrBayes v. 3.2.7 [26]. A total of 5,000,000 generations were run, sampled every 500 generations, with four independent runs and four chains. The first 25% of samples from the cold chain were discarded as burn-in. Trees were visualized in FigTree v. 1.4.4 [27].

### 2.3. Riboprobe Synthesis

The plasmids with DNA templates were linearized to generated riboprobes using the DIG RNA labeling KIT SP6/T7 (Roche, Basel, Switzerland), following the manufacturer’s protocol. Riboprobes purified with MEGAclearTM Transcription clean-up kit (Ambion, Kaufungen, Germany) were used at the working concentration of 0.5 ng/μL during hybridization. Probes were denatured at 80 °C to 90 °C for 10 min before hybridization.

### 2.4. Regeneration and In Situ Hybridization

Animals 80–120 mm in length were selected for amputation/regeneration experiments. They were starved for 2 weeks to gain a low level of alkaline phosphatase. During this period, the worms were kept in the dark at 14 °C [28]. After being relaxed in 1:1 mixture of 7.5% MgCl_2_ and seawater, worms were cut into two pieces behind the mouth (Figure S1A, Supplementary Materials). Then, the anterior and the posterior body fragments were incubated separately in 12 cm petri dishes under the aforementioned conditions. The first batch of operated animals were used to examine the regenerative time course using an Olympus BX53 microscope adapted with a DP72 camera. According to results of this observation, in situ hybridization samples were collected at 0 (intact specimens), 0.5, 1.5, 2, 4, 6, 10, 14, 20, 26, 32, and 40 dpa (days post amputation) from the later batches of experimental animals.

After being relaxed with MgCl_2_ (see above) for 30 min, 0 dpa (intact) worms were cut into fragments each about 1 cm in length, and then fixed for in situ hybridization experiment. For animals undergoing posterior regeneration, the whole regenerating worms/fragments were fixed for in situ hybridization. For animals undergoing anterior regeneration, the anterior 1 cm fragments and fragments of the intestinal region were selected for in situ hybridization; the residuals of some specimens were fixed for a paraffin section.

Specimens for in situ hybridization were fixed in 4% PFA (paraformaldehyde) in PBS for 20 min, followed by 10% *N*-acetyl cysteine (NAC; Sigma, St. Louis, MO, USA) solution for 20 min to remove their mucus. Then, the samples were transferred into the Fixative overnight at 4 °C. The fixed samples were dehydrated by washing for 10 min in 25%, 50%, 75% (*v*/*v*) methanol in PBS, and 100% methanol, and stored at −20 °C for at least 2 h. The bleached samples were incubated in a 5% H_2_O_2_/methanol medium until pigmentation completely disappeared. Whole-mount in situ hybridization was prepared following published protocols [28,29]. Worms were treated in 10 mg/mL proteinase K with 0.2% SDS for 10 min at 37 °C, followed by a fixation for 30 min. In order to allow probe penetration, the samples were permeabilized with reduction buffer (50 mM DTT, 1% NP-40, 0.5% SDS, in 1× PBS) for 10 min with intermittent gentle agitation at 37 °C. Worms were treated in the hybridization solution (containing 50% formamide, 5× SSC, 1 mg/mL yeast RNA, 100 μg/mL heparin, 0.05% Triton-TX, 0.05% Tween-20, 10 mg/mL sperm DNA) for 3 h at 57 °C. The solution was replaced with fresh hybridization solution containing the probe (*Ls-piwi1* and *Ls-pcna* 0.2 μg/mL; *Ls-piwi2* and *Ls-piwi3* 1.5 μg/mL), and specimens were incubated at 57 °C for at least 16 h. The hybridized specimens were washed twice in wash buffer 1 (50% formamide, 5× SSC, 0.1% TritonX-100) at 57 °C for 20 min each, washed twice in wash buffer 2 (2× SSC, 0.1% TritonX-100) at 57 °C for 15 min each, and washed thrice in wash buffer 3 (0.1× SSC, 0.1% TritonX-100) at 57 °C for 20 min each. Washed specimens were incubated at 30 °C for 4 h in blocking solution containing 2% blocking reagents, 1% goat serum (Roche) in MABT (100 mM maleic acid, 150 mM NaCl, 0.1% Tween-20, adjusting pH to 7.5 with NaOH), and then incubated at 4 °C overnight with 1/5000 anti-DIG/AP antibody (Roche) in blocking solution. After incubation, the specimens were washed six times at 30 °C in MABT for 20 min each, and color reaction was performed with BM Purple (Sigma) containing 5 mM levamisole in the dark. After 4% PFA fixation for 20 min, the specimens were treated with 100% ethanol for 30 min to remove nonspecific background staining. Then, stained samples were washed in PBS and mounted on slides in 80% glycerol in PBS. Photomicrographs were obtained using an Olympus SZX 16 stereomicroscope adapted with a DP74 camera. At least three specimens were examined for each gene and stage.

Previous studies documented that starvation might cause the loss of gene expression signal as quickly as 1 week after starvation (e.g., [30]). To determine the potential effect of starvation on expressions of the studied genes, we also carried out in situ hybridization experiments using non-starved worms (intact worms only; 3 days after feeding).

### 2.5. Histology

Samples for paraffin sections were fixed in the Bouin’s solution and then dehydrated in an ascending series of ethanol (70%, 80%, 95%, and twice 100% ethanol). After that, the samples were cleared in xylene and embedded in paraffin; next, 7 μm sections were made using a BM 2016 rotary microtome (Leica, Wetzlar, Germany). The sections were stained with the Mallory triple staining method.

In situ hybridization positive samples for frozen sections were dehydrated overnight in 30% sucrose in PBS and then sectioned to 12 μm using a CM 1850 Microtome Cryostat (Leica). After hydrating in PBS, the sections were observed and photographed using an Olympus BX 53 microscope adapted with a DP72 camera.

## 3. Results

### 3.1. Characterization of Argonaute Family in Lineus sanguineus and Notospermus geniculatus

**Figure 1:**
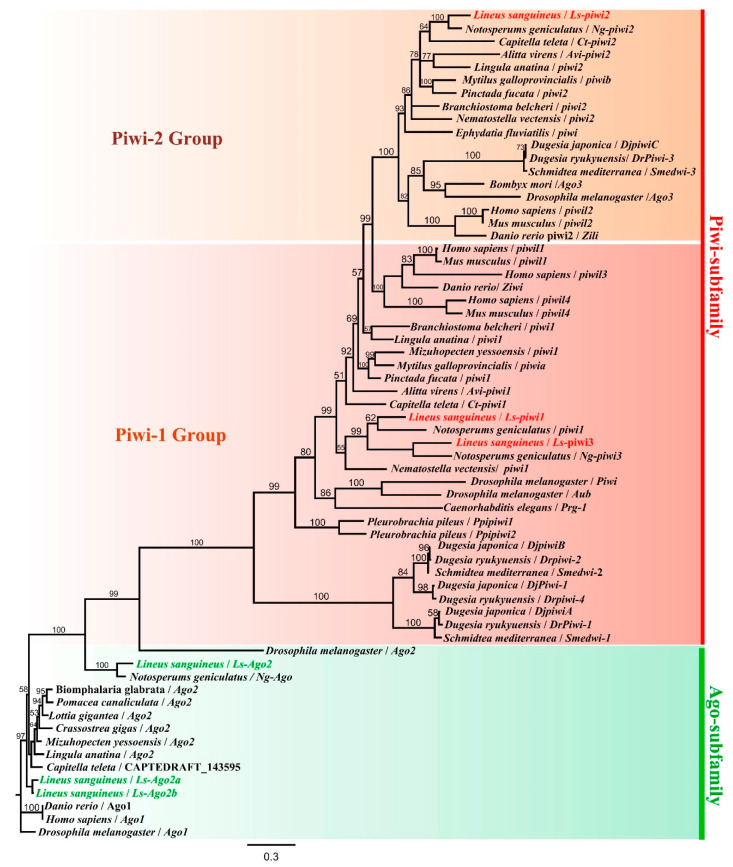
Bayesian consensus tree of Argonaute proteins of *Lineus sanguineus* and other metazoans. Posterior probabilities are shown by the numbers above the nodes.

BLASTp searches of the *Lineus sanguineus* genomic library revealed six candidate homologs of the *Drosophila melanogaster* Piwi gene. All of them contained the characteristic PAZ (PIWI-Argonaute-Zwille) and PIWI domains of the Argonaute family (Figure S2, Supplementary Materials), which were highly conserved among animals [31,32]. Three homologs (*Ls-piwi1*, *Ls-piwi2*, and *Ls-piwi3*), which possessed only PAZ and PIWI domains and could be translated into proteins containing 886, 944, and 970 amino-acid residues, respectively (Figure S2, Supplementary Materials), were located in a unique clade representing the Piwi subfamily (Figure 1). The other three (*Ls-Ago2*, *Ls-Ago2a*, and *Ls-Ago2b*), which were translated into 1179, 826, and 899 amino-acid residues, respectively, also contained the N-terminal and the MID (middle) domains (Figure S2, Supplementary Materials) and accordingly belonged to the Ago subfamily [10,31]. Four Argonaute proteins were found from the *Notospermus geniculatus* protein library, including three PIWI proteins (*Ng-piwi1*, *Ng-piwi2*, and *Ng-piwi3*) and one AGO protein (*Ng-Ago2*) (Figure S2, Supplementary Materials). Phylogenetic analysis showed that each of the three *piwi* genes of *N. geniculatus* clustered together with the corresponding genes of *L. sanguineus* (Figure 1). The nomenclature of these nemertean proteins was based either on the position in phylogenetic tree (*Ls-piwi2* and AGOs; Figure 1) or on their identities to *D. melanogaster* PIWI protein (*Ls-piwi1*, 43.1%; *Ls-piwi3*, 40.1%).

A *pcna* homolog was identified from the *L. sanguineus* genome. It encoded a protein containing 259 amino acids, which had a 92% similarity with *N. geniculatus*. Its similarity with the PCNA proteins of nine other metazoans ranged from 47.1% (*Dugesia japonica*) to 55.9% (*Xenopus laevis* and *Homo sapiens*), and all shared 74 conservative sites (Figure S3, Supplementary Materials).

### 3.2. Regenerative Time Course of Lineus sanguineus

The regeneration scheme of L. sanguineus was previously documented in detail [4,33], and our results were basically in accordance with previous studies. After amputation, the anterior fragment underwent posterior regeneration, while the posterior fragment underwent anterior regeneration. They both healed the wound within 2 dpa (Figure S1B,B’, Supplementary Materials). Then, the posterior fragment formed a blastema which grew bigger but did not differentiate recognizable organs during the period of 4–14 dpa (Figure S1C’,D’,E’,F’, Supplementary Materials). The paired cerebral ganglia and cerebral organs became distinct at 20 dpa (Figure S1G’, Supplementary Materials). At this stage, the new mouth formed at the junction of new and old tissues; the new proboscis, as well as the rhynchocoel, was visible between the paired cerebral ganglia (Figure S1G’, Supplementary Materials). Then, the rhynchocoel extended posteriorly to join up with the original remnant rhynchocoel (Figure S1H’, Supplementary Materials). Thus, a new and complete rhynchocoel formed. Ocelli, which represented the accomplishment of anterior regeneration, usually appeared at 40 dpa (Figure S1J’, Supplementary Materials).

The posterior regeneration of the anterior fragment involved the reconstruction of a new gut. Between 4 and 10 dpa, the regenerative fragment elongated and tapered posteriorly, while the proboscis extended posteriorly (Figure S1C–E, Supplementary Materials). The arising of the intestine was first detected by 14 dpa (Figure S1F, Supplementary Materials), and a distinct intestine behind the stomach could be seen by 20 dpa (Figure S1G, Supplementary Materials). By 26 dpa and later stages, the body became slenderer and gradually restored the normal proportion of different body regions (Figure S1H–J, Supplementary Materials).

### 3.3. Expression Patterns of piwi and pcna Genes in Intact Worms

**Figure 2:**
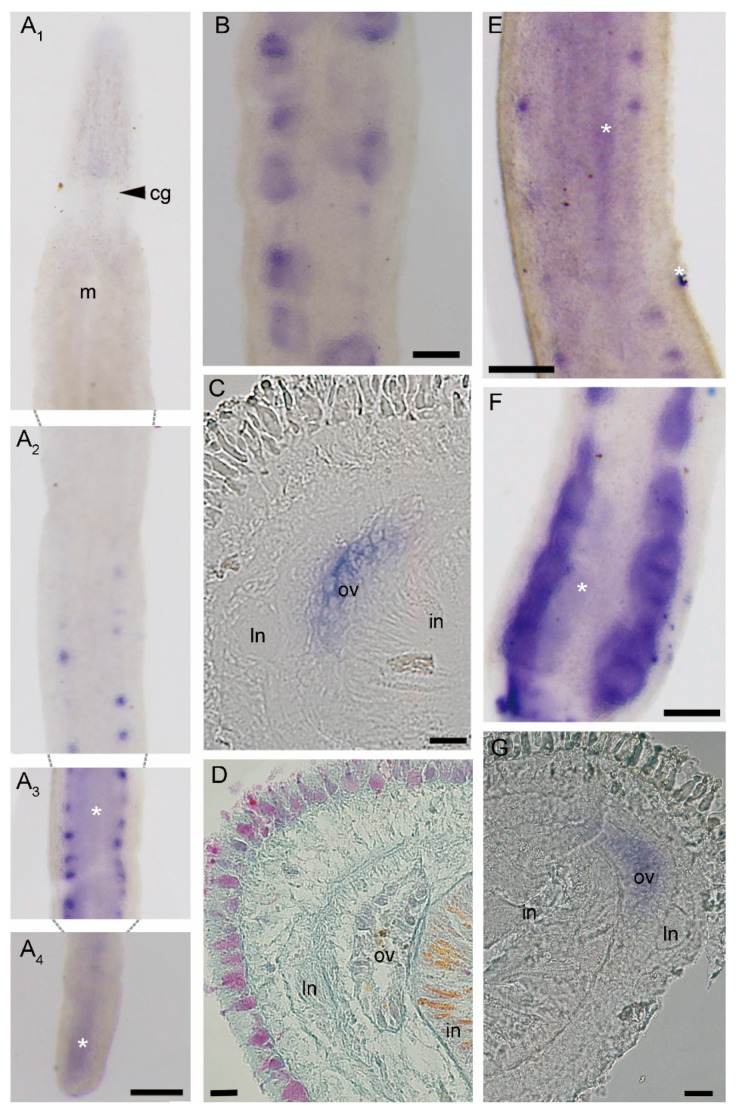
Expression patterns of *Ls-piwi1* and *Ls-pcna* (proliferating cell nuclear antigen) in intact worm of *Lineus sanguineus*. (**A**) *Ls-piwi1* expression in an intact worm, showing it is not expressed in the anterior body region (from head to anterior intestinal region) (**A_1_**,**A_2_**) and the caudal end (**A_4_**), and is only expressed in ovaries that are distributed in the intestinal region (**A_2_**,**A_3_**). (**B**) *Ls-piwi1* expression in an individual with more developed ovaries. (**C**) Transverse frozen section of a *Ls-piwi1* in situ hybridized specimen through the region of (**A_3_**). (**D**) Transverse section of intestinal region (Mallory trichrome staining) showing the shape and position of ovaries. (**E**,**F**) *Ls-pcna* expression in two different individuals with ovaries at different developmental stages. (**G**) Transverse frozen section from (**E**). Abbreviations: cg, cerebral ganglia; in, intestine; ln, lateral nerve; m, mouth; ov, ovary. Asterisks mark nonspecific staining (unexpected spots on body surface and diffuse staining in intestine; see Figure S4, Supplementary Materials). Scale bars: (**A**,**B**,**E**,**F**) = 200 μm; (**C**,**D**,**G**) = 20 μm.

**Figure 3:**
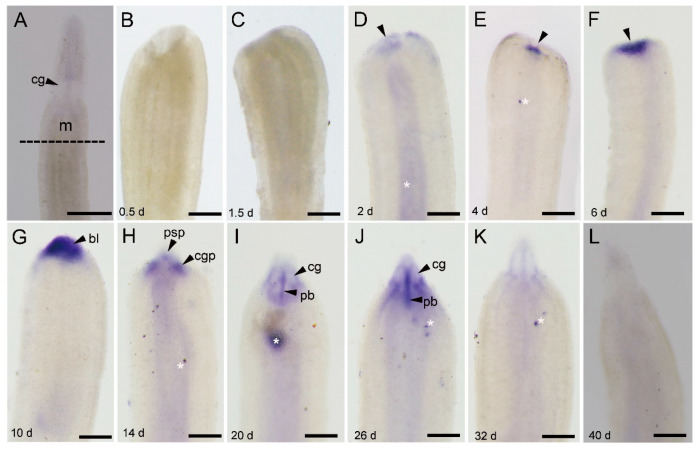
*Ls-piwi1* expression during the anterior regeneration of *Lineus sanguineus*. (**A**) Anterior region of an adult worm (control). The dashed line indicates the site to perform amputation. (**B**,**C**) Images from 0.5 and 1.5 days post amputation (dpa), respectively, no positive staining. (**D**) Image from 2 dpa, with complete wound healing and slight expression (arrowed). (**E**–**G**) Images from 4, 6, and 10 dpa, respectively, showing expression in the blastema (arrowed). (**H**) Image from 14 dpa, showing expression in three parts, an anterio-middle part and two posterio-lateral parts. (**I**,**J**) Images from 20 and 26 dpa, respectively, with expression in the proboscis and around cerebral ganglia. (**K**) Image from 32 dpa, with weak expression around cerebral ganglia. (**L**) Image from 40 dpa, no positive expression. Abbreviations: cg, cerebral ganglia; cgp, cerebral ganglion primordium; m, mouth; pb, proboscis; psp, proboscis and proboscis sheath primordium. Asterisks mark nonspecific staining (unexpected spots on body surface and diffuse staining in intestine; see Figure S4, Supplementary Materials). Scale bars: (**A**) = 500 μm; (**B**–**L**) = 200 μm.

**Figure 4:**
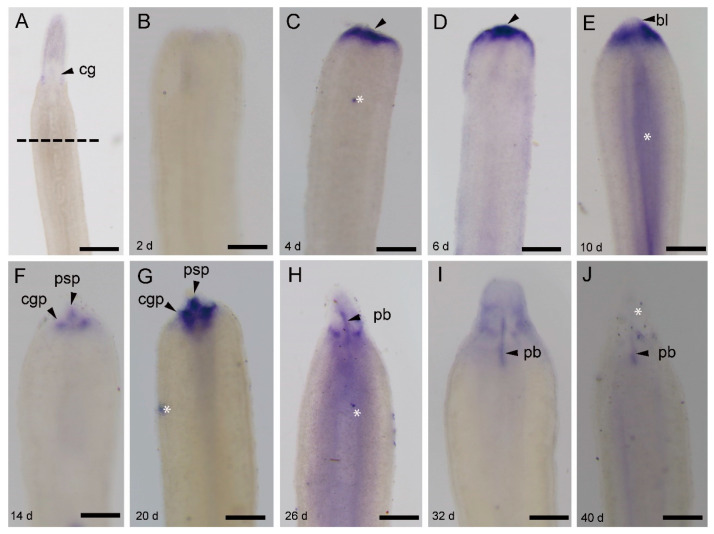
*Ls-pcna* expression during the anterior regeneration of *Lineus sanguineus*. (**A**) Anterior region of an adult worm (control); the dashed line indicates the site to perform amputation. (**B**) Image from 2 dpa, no expression. (**C**–**E**) Images from 4, 6, and 10 dpa, respectively, expressing in the blastema (arrowed). (**F**,**G**) Images from 14 and 20 dpa, respectively, with expression in three parts, an anterio-middle part and two posterio-lateral parts. (**H**,**I**) Images from 26 and 32 dpa, respectively, with expression in proboscis and around cerebral ganglia. (**J**) Image from 40 dpa, with expression only in proboscis (arrowed). Abbreviations: bl, blastema; cg, cerebral ganglion; cgp, cerebral ganglion primordium; pb, proboscis; psp, proboscis and proboscis sheath primordium. Asterisks mark nonspecific staining (see Figure S4, Supplementary Materials). Scale bars: (**A**) = 500 μm; (**B**–**J**) = 200 μm.

**Figure 5:**
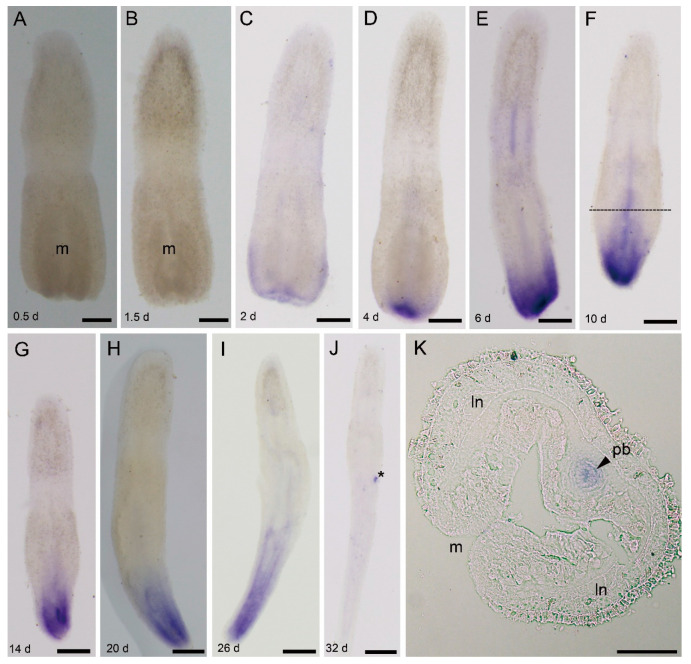
*Ls-piwi1* expression during the posterior regeneration of *Lineus sanguineus*. (**A**,**B**) Images from 0.5 and 1.5 dpa, respectively, no positive staining. (**C**) Image from 2 dpa, weak expression around the wound site. (**D**) Image from 4 dpa, expressing at the center of the wound. (**E**) Image from 6 dpa, expressing in the posterior end and the proboscis. (**F**) Image from 10 dpa, expressing in the posterior end and proboscis (dashed line roughly showing the site of frozen section, see (**K**)). (**G**–**I**) Images from 14, 20, and 26 dpa, respectively, expressing in the posterior end. (**J**) Image from 32 dpa, no positive staining. (**K**) Frozen section of a 10 dpa in situ hybridized specimen (same specimen as (**F**)), showing the expression in proboscis. Abbreviations: ln, lateral nerve; m, mouth; pb, proboscis. Asterisks mark nonspecific staining (see Figure S4, Supplementary Materials). Scale bars: (**A**–**J**) = 250 μm; (**K**) = 50 μm.

**Figure 6:**
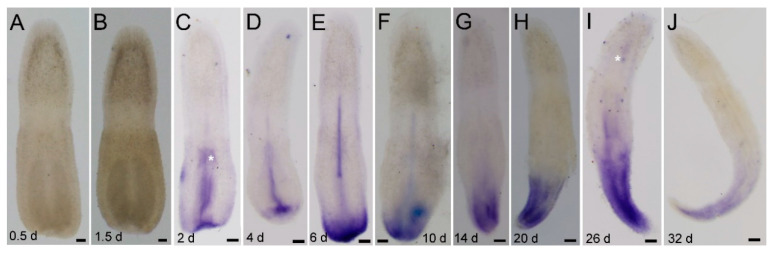
*Ls-pcna* expression during the posterior regeneration of *Lineus sanguineus*. (**A**,**B**) Images from 0.5 and 1.5 dpa, respectively. (**C**) Image from 2 dpa, expressing in the wound and proboscis. (**D**) Image from 4 dpa, expressing in proboscis and near the posterior end. (**E**,**F**) Images from 6 and 10 dpa, respectively, expressing in the posterior end and proboscis. (**G**–**J**) Images from 14, 20, 26, and 32 dpa, respectively, expressing at the posterior end. Asterisks mark nonspecific staining (see Figure S4, Supplementary Materials). Scale bars = 100 μm.

**Figure 7:**
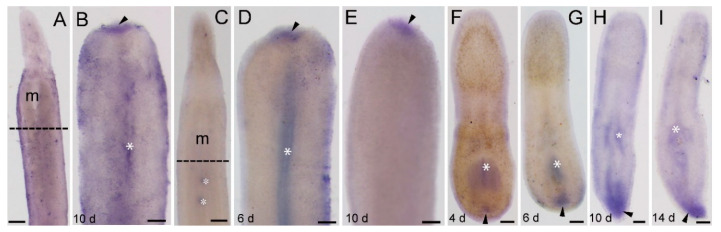
Expression of *Ls-piwi2* and *Ls-piwi3* during the regeneration of *Lineus sanguineus*. (**A**) Anterior region of an adult worm (control of *Ls-piwi2*); the dashed line indicates the site to perform amputation. (**B**) *Ls-piwi2* expression during anterior regeneration (10 dpa; arrowed). (**C**) Anterior region of an adult worm (control of *Ls-piwi3*); the dashed line indicates the site to perform amputation. (**D**,**E**) *Ls-piwi3* expression during anterior regeneration (6 and 10 dpa, respectively; arrowed). (**F**–**I**) *Ls-piwi3* expression during posterior regeneration (4, 6, 10, and 14 dpa, respectively; arrowed). Expression domains are arrowed. Asterisks mark nonspecific staining (see Figure S4, Supplementary Materials). Scale bars: (**A**,**C**) = 250 μm; (**B**,**D**–**I**) = 100 μm.

When using the sense probes of the four genes studied, unspecific staining was detected in the blood lacuna behind the cerebral ganglia, the intestine, and the rhynchocoel, and unexpected spots were observed on the body surface (Figure S4, Supplementary Materials). Such unspecific signals appeared also in antisense probe hybridized samples in the following experiments (Figure 2, Figure 3, Figure 4, Figure 5, Figure 6 and Figure 7).

In intact worms, *Ls-piwi1* was expressed only in the intestinal region. Positive reactions of in situ hybridization exhibited blocks displayed as two rows along the lateral sides, where their sizes varied greatly among individuals (Figure 2A,B). Frozen sections showed that they were located near the lateral nerves and outside the intestine (Figure 2C). Mallory trichrome staining for paraffin sections showed that the structures at the same position were ovaries (Figure 2D).

With distinct signals appearing in ovaries, the expression pattern of *Ls-pcna* (Figure 2E–G) was similar to that of *Ls-piwi1*. We did not detect any expression of *Ls-piwi2* and *Ls-piwi3* in intact worms. In situ hybridization experiments with non-starved animals exhibited the same results (i.e., *Ls-pcna* and *Ls-piwi1* expressing in ovaries; *Ls-piwi2* and *Ls-piwi3* not expressing) (Figure S5, Supplementary Materials), indicating that expression patterns of these genes were not obviously impacted by a 14 day starvation.

### 3.4. Expression of piwi and pcna Genes during Regeneration

#### 3.4.1. *Ls-piwi1* and *Ls-pcna* Expression during Anterior Regeneration

At all stages of anterior regeneration, *Ls-piwi1* was expressed to the same extent in the pre-existing gonads as that in intact worms.

The expression of *Ls-piwi1* was not detected at the wound site until 2 dpa (Figure 3B–D). Then the expression became stronger with the growing of blastema at 4 to 10 dpa (Figure 3E–G). By 14 dpa, the expression area divided into three parts, an anterio-middle part and two posterio-lateral parts (Figure 3H), with the anterio-middle one representing the expression in the proboscis and proboscis sheath primordium (psp), and paired posterio-lateral ones representing the expression in the cerebral ganglia primordium (cgp) [5,33]. At later stages (20, 26, 32 dpa) when the cerebral ganglia were recognizable, positive expression was detected around the cerebral ganglia (Figure 3I–K). By 20 and 26 dpa, there was a distinct expression in the regenerating proboscis, which showed a longitudinal band between left and right cerebral ganglia (Figure 3I,J). We did not find any expression of *Ls-piwi1* in the pre-existing tissues of the old rhynchocoel. By 40 dpa, when the anterior regeneration accomplished, *Ls-piwi1* expression was not observed (Figure 3L).

The expression pattern of *Ls-pcna* (Figure 4) was similar to that of *Ls-piwi1*. A visible difference was that *Ls-pcna* was still expressed in the new proboscis at 40 dpa (Figure 4J), while *Ls-piwi1* was not detectable at this stage (Figure 3L).

#### 3.4.2. *Ls-piwi1* and *Ls-pcna* Expression during Posterior Regeneration

During posterior amputation, expression of *Ls-piwi1* was first detected by 2 dpa near the wound (Figure 5A–C). As regenerating tissues grew (4 to 14 dpa), the expression scope expanded, and the density became stronger (Figure 5D–G). The expression of *Ls-piwi1* in the remnant proboscis was conspicuous at 6 and 10 dpa (Figure 5E,F,K). Afterward (14 to 26 dpa), *Ls-piwi1* was continuously expressed in the posterior end, while no expression was observed in the proboscis (Figure 5G–I). By 32 dpa, no positive signals were detected in the whole body (Figure 5J).

The expression pattern of *Ls-pcna* (Figure 6) was similar to that of *Ls-piwi1*. Differences observed include (1) strong expression in the hind end appearing earlier (2 dpa; Figure 6C) than for *Ls-piwi1* (2 dpa; Figure 5C), (2) expression in the remnant proboscis appearing earlier (2–10 dpa; Figure 6C–F) than for *Ls-piwi1* (6, 10 dpa; Figure 5E,F), and (3) continued expression by 32 dpa (Figure 6J) when *Ls-piwi1* showed no positive signals (Figure 5J).

### 3.5. Ls-piwi2 and Ls-piwi3 Expression during Regeneration

During anterior regeneration, *Ls-piwi2* and *Ls-piwi3* were only expressed weakly in the blastema, detectable by 10 dpa (Figure 7B) and by 6 and 10 dpa (Figure 7D,E), respectively. At later stages, no expression was detected for these genes.

During posterior regeneration, *Ls-piwi3* was expressed at the posterior end from 4 to 14 dpa (Figure 7F–I). A positive reaction was not detected for *Ls-piwi2*.

## 4. Discussion

Members of the Argonaute protein family, defined by the conservative PAZ and PIWI domains, are found in almost all organisms including bacteria, archaea, and eukaryotes [34,35]. The number of Argonaute genes varies from 1 in the fission yeast *Schizosaccharomyces pombe* to 27 in the nematode *Caenorhabditis elegans* [36,37]. Their numbers are often not conservative even among closely related animals. For instance, five Argonaute members are found in *Drosophila melanogaster* [38], while we identified only three Argonaute genes in the genome of *Bombus vancouverensis* (NCBI Accession Number, PRJNA623917). This is also the case for phylum Nemertea, where two heteronemertean species, *L. sanguineus* and *N. geniculatus*, have six (three *piwi* and three *ago*) and four (three *piwi* and one *ago*) Argonaute genes, respectively (Figure S2, Supplementary Materials).

As shown in Figure 1, some *piwi* genes from the same animal group, such as *Ls-piwi1* and *Ls-piwi3* of nemerteans, *Piwil1* and *Piwil3* of mammals, and *Smedwi-1*, *Smedwi-2*, *DjpiwiA*, *DjpiwiB*, and *Djpiwi1* of planarians, are clustered together in the phylogenetic tree, suggesting that *piwi* numbers of different animal groups might increase independently via gene duplication. Since most animal phyla have at least two *piwi* genes, it is likely that two *piwi* genes evolved in earlier ancestors of metazoans, with *piwi2* being derived from *piwi1*.

The nomenclature of Argonaute family members is chaotic. For example, among the five Argonaute genes of *D. melanogaster* (*Piwi*, *Aub*, *Ago1*, *Ago2*, and *Ago3*), *Aub* and *Ago3* are two *piwi* genes belonging to Piwi-1 group and Piwi-2 group, respectively; the *piwi2*/*piwiB* of three turbellarian species is not located in the Piwi-2 group, while their *piwi3*/*piwiC* is clustered in the Piwi-2 group in the phylogenetic tree (Figure 1). In the present study, the nomenclature of nemertean *piwis* was mainly based on their position in the tree.

Studies with several model animals (e.g., *D. melanogaster*, *C. elegans*, *Mus musculus*, and *Daphnella ryukyuensis*) have demonstrated that they play important roles in germline development, whereby mutation or RNAi of some *piwi* genes might cause sterile or even lethal effects (e.g., [8,39,40,41]). Our results show that *Ls-piwi1* is expressed in ovaries of intact worms and amputated posterior body fragments of *L. sanguineus* (Figure 2A–C), while expressions of *Ls-piwi2* and *Ls-piwi3* are not observed in ovaries. In *D. melanogaster*, all three *piwi* genes, *Piwi* (Piwi-1 group), *Aub* (Piwi-1 group), and *Ago3* (Piwi-2 group) (Figure 1), are expressed in female and male germline cells [8,42,43,44]. The *Prg-1* (Piwi-like 1) of *C. elegans* is expressed in male germ cells [40]. *Ziwi* (Piwi-like 1) and *Zili* (Piwi-like 2) of *Danio rerio* are expressed in both female and male gonads [14,45]. The three *piwi* genes of *M. musculus*, *Miwi* (Piwi-like 1), *Mili* (Piwi-like 2), and *Miwi2* (Piwi-like 4), are expressed only in male gonads [39,46,47]. The *Ct-piwi1* of *Capitella teleta* (Annelida) is expressed in both female and male gonads [48]. In *D. ryukyuensis*, all four *piwi* genes (*Dr-piwi1*, *Dr-piwi2*, *Dr-piwi3*, and *Dr-piwi4*) are expressed in male and female gonads [41]. The *Hywi* (Piwi-like 1) and *Hyli* (Piwi-like 2) of *Hydra* are expressed in female gonads [49]. The *PpiPiwi1* and *PpiPiwi2* of *Pleurobrachia pileus* (Ctenophora) are expressed in both male and female gonads [16]. These results suggest a great variation in the germline expression of *piwi* genes among different animals. Except for *C. elegans* and *M. musculus*, the *piwi1* of all aforementioned animals is expressed in female gonads. The non-*piwi1* genes (i.e., *Ls-piwi2* and *Ls-piwi3*) of *L. sanguineus* seem not to function in the female germline development. This is different from *Drosophila*, *Dugesia*, *Hydra*, and *Pleurobrachia*; however, *L. sanguineus* is not the only animal species with these genes silent in female gonads. For example, the *Ta-piwi2* of *Typosyllis antoni* (Annelida) is expressed in male testes but not in female ovaries [50]. Unfortunately, no development of testes was observed in our experimental animals; thus, whether *piwi* genes of *L. sanguineus*, particularly *Ls-piwi2* and *Ls-piwi3*, are expressed in male germlines remains to be studied.

*Piwi* genes, essential for stem-cell functions, are expressed in stem cells in many organisms. Animals with the ability to regenerate their whole body express *piwi* genes in somatic stem cells; the two major metazoan clades lacking this ability (ecdysozoans and vertebrates) lack somatic stem cells with *piwi* expression [51,52]. In invertebrates, some types of cells that express *piwi* genes and are responsible for whole-body regeneration are thought to be stem cells or with characteristics of stem cells, e.g., the archeocytes and choanocytes of the demosponge *Ephydatia fluviatilis* [53], the interstitial cells (i-cells) of *Hydra* [49,54], and the neoblasts of *D. japonica* [55]. The present study did not detect any *piwi* expressions in somatic tissues of intact *L. sanguineus* (Figure 2A). Turbeville’s histological study did not find any cells morphologically identical to stem cells within the extracellular matrix of this nemertean [7]. Given that the whole-mount in situ hybridization may not resolve the gene expression in single cell, we cannot conclude here that adult *L. sanguineus* possess no somatic stem cells, but they, if any, should be rare. In regenerating (anterior and posterior) body fragments, *Ls-piwi1* and *Ls-pcna* were expressed by 2 or 4 dpa (Figure 3, Figure 4, Figure 5 and Figure 6). Their strong expressions in the blastema (Figure 3E–G; Figure 4C–E) suggest aggregation and active proliferation of some kind(s) of “pluripotent cells”. The origin of these cells remains an unanswered question, but they are more likely to be dedifferentiated from other cells near the wound than congenital stem cells. This hypothesis seems in accordance with that documented for the polychaete *Platynereis dumerilii*, whose regenerative blastema cells are mostly derived from cells in the segment immediately adjacent to the amputation site [56]. Another example of cell dedifferentiation is in the limb regeneration of Mexican axolotl *Ambystoma mexicanum*, whose blastemal cells are largely derived from somatic cells including multinucleated muscle cells, epidermal basal keratinocytes, and fibroblasts in dermal and connective tissues [57]. The cell dedifferentiation hypothesis in the regeneration of *L. sanguineus* surely needs to be confirmed by future cell mark and tracing experiments.

During both anterior and posterior regeneration of *L. sanguineus*, wound healing completed in 2 days. No expressions of *Ls-piwi1* and *Ls-pcna* were observed at 0.5 and 1.5 days (Figure 3B,C, Figure 5A,B, and Figure 6A,B), indicating the lack of “pluripotent cells” and cell proliferation during this period. This supports a previous hypothesis that wound healing is accomplished by cellular migration rather than by cell proliferation in this nemertean [5]. During the blastema stage (2–10 dpa) of anterior regeneration, the strong expressions of *Ls-piwi1* and *Ls-pcna* suggest that this may be a stage for preparing “pluripotent cells”, probably via dedifferentiation and proliferation. While the blastema differentiates into three primordia (14 dpa), the expression of *Ls-piwi1* is divided into three corresponding parts (Figure 3H). Then, when cerebral ganglia form (20, 26 dpa), *Ls-piwi1* expression becomes indetectable in the differentiated cerebral ganglia but is expressed in tissues around cerebral ganglia (Figure 3I,J), which might be related to the formation of sensory organs such as cerebral organs, ocelli, and cephalic slits. After these main organs of the head and a new proboscis apparatus are differentiated, *Ls-piwi1* ceases to be expressed in the regenerating tissues (Figure 3L). Similarly, during posterior regeneration *Ls-piwi1* ceases to be expressed when the reconstruction of a new intestine finished (Figure 5J). By contrast, *Ls-pcna* continues to be expressed at later stages of regeneration (Figure 4J and Figure 6J). These results suggest that the differentiation/formation of regenerating organs may be dependent on *Ls-piwi1* expression, and the proliferation of differentiated cells may continue to contribute to the growth of the regenerated organs. The necessity of *piwi* expression in the differentiation of neoblasts was proven in a previous study on *Dugesia japonica* (*Dj-piwiB*) [58].

In addition to *Ls-piwi1*, *Ls-piwi2* and *Ls-piwi3* are also expressed during anterior regeneration, and the latter is also expressed during the posterior of *L. sanguineus*, whereas they are expressed only at the blastema stage of anterior regeneration or similar early stage of posterior regeneration. For the turbellarian *S. mediterranea*, worms are unable to regenerate if *Smedwi-2* or *Smedwi-3* (belonging to Piwi-1 and Piwi-2 groups, respectively; see Figure 1) is inhibited [19]. In *D. ryukyuensis*, RNAi of *Dr-piwi2* or *Dr-piwi3* results in failure of regeneration [41]. In the acoelan *Hofstenia miamia*, dividing cells express transcripts of two *piwi* homologs, and *piwi-1* is required for regeneration [59]. In the urochordate *Botryllus leachi*, inhibition of *BlPiwi* results in a complete halt of whole-body regeneration [60]. For non-whole-body regeneration, a well-studied example is the limb regeneration of the Mexican axolotl *A. mexicanum*, in which both *piwi-like 1* and *piwi-like 2* are expressed in the blastemal cells, the basal layer keratinocytes, and the thickened apical epithelial cap in the wound epidermis [57]. Within Lophotrochozoa *s. str.* (e.g., [61]), regeneration and *piwi* expression are well studied only for several annelid species such as *Capitella teleta*, *Platynereis dumerilii*, and *Alitta virens*. In these polychaetes, both *piwi1* and *piwi2* genes are expressed in the blastema and posterior growth zone during (posterior) regeneration, but are not expressed during the wound healing stage [48,56,62]. This is similar to that observed in the regeneration of *L. sanguineus*. Annelids have a segmented body and a posterior growth zone, and their *piwi* expression is restricted to the posterior growth zone [48]. The *Ls-piwi1* of *L. sanguineus* also has a near caudal end expression during later stages of posterior regeneration (Figure 5G–I). Knowledge on the growth of nemerteans is limited. An early study documented three types of growth: growth by enlargement, growth by addition of similar parts, and growth by replacement (of some structures such as stylet apparatus) [63]. Among them, the “growth by addition of similar parts”, which is characterized by the body increasing in length via the symmetrical formation of additional similar parts (being “essentially budding in which the new parts remain and become functionally integrated with the old”) [63], is similar to the growth of annelids. In this case, the caudal region of nemerteans may function similarly to the posterior growth zone of annelids.

## 5. Conclusions

In conclusion, *L. sanguineus* has three *ago* and three *piwi* genes. None of the three *piwis* are expressed in somatic tissues of intact worms, and only *Ls-piwi1* is expressed in gonads of the female. All three *piwi* genes are expressed during the regeneration of this nemertean, except that the expression of *Ls-piwi2* is not detected during posterior regeneration. No direct evidence was found for the existence of congenital stem cells in *L. sanguineus*, where the pluripotent cells in regenerating tissues are likely to be dedifferentiated from other cells. Future studies may focus on types of cells contributing to stem cells and blastema, gene functions, and the *piwi* expression patterns of other nemerteans including species unable to regenerate.

## Supplementary Materials

The following are available online at https://www.mdpi.com/2073-4425/11/12/1484/s1: Table S1: Species and their GenBank accession numbers of Argonaute protein family used for phylogenetic analysis; Figure S1: Time course of the regeneration of *Lineus sanguineus* (ventral view); Figure S2: Domain structures of the inferred Argonaute proteins in *Lineus sanguineus* and *Notospermus geniculatus*; Figure S3: A comparison of the deduced amino-acid sequences of the proliferating cell nuclear antigen (PCNA); Figure S4: Micrographs for sense-probe staining (control) of four genes in *Lineus sanguineus*; Figure S5: Expression patterns of *Ls-pcna*, *Ls-piwi1*, *Ls-piwi2*, and *Ls-piwi3* in the intestinal region of non-starved *Lineus sanguineus*.
