# Supplementary material for: Expression of Piwi Genes during the Regeneration of Lineus sanguineus (Nemertea, Pilidiophora, Heteronemertea)
Source: Genes (Basel). 2020 Dec 10;11(12):1484. doi: 10.3390/genes11121484 (PMC7764242; doi:10.3390/genes11121484)
Supplement: Supplementary file 1 [file genes-11-01484-s001.pdf]

Supplementary materials

Table S1. Species and their GenBank accession numbers of Argonaute protein family used for phylogenetic analysis.

Species and gene	Accession number	Species and gene	Accession number
Drosophila melanogaster Ago1	NP_725341.1	*Branchiostoma belcheri* Piwi1	XP_019620713.1
Homo sapiens Ago1	NP_036331.1	*Mus musculus* Piwil4	BAF65667.1
Danio rerio Ago1	AFU66007.1	*Homo sapiens* Piwil4	NP_689644.2
Capitella teleta CAPTEDRAFT_143595	ELT99790.1	*Danio rerio* Piwi1	NP_899181.1
Lingula anatina Ago2	XP_013421307.1	*Homo sapiens* Piwil3	BAC81343.1
Mizuhopecten yessoensis Ago2	OWF49186.1	*Mus musculus* Piwil1	NP_067286.1
Crassostrea gigas Ago2	XP_011417884.1	*Homo sapiens* Piwil1	NP_004755.2
Lottia gigantea Ago2	XP_009064000.1	*Schmidtea mediterranea* Piwi3	ACC97187.1
Pomacea canaliculata Ago2	XP_025092978.1	*Bombyx mori* Ago3	BAF98575.1
Biomphalaria glabrata Ago2	XP_013068459.1	*Danio rerio* Piwi2	ACF35261.1
Schmidtea mediterranea Piwi2	ABB77338.1	*Mus musculus* Piwil2	NP_060538.2
Schmidtea mediterranea Piwi1	ABB77337.1	*Homo sapiens* Piwil2	NP_067283.1
Drosophila melanogaster Aub	AGA18946.1	*Nematostella vectensis* Piwi2	ASW22511.1
Drosophila melanogaster Piwi	AAD08705.1	*Ephydatia fluviatilis* Piwi	BAJ07609.1
Nematostella vectensis Piwi1	ASW225 10.1	*Branchiostoma belcheri* Piwi2	XP_019639428.1
Alitta virens Piwi1	AJW77405.1	*Pinctada fucata* Piwi2	QAW56457.1
Pinctada fucata Piwi1	QAW56456.1	*Mytilus galloprovincialis* Piwib	AMN88362.1
Mytilus galloprovincialis Piwia	AMN88361.1	*Lingula anatina* Piwi2	XP_013382739.1
Mizuhopecten yessoensis Piwi1	XP_021371712.1	*Alitta virens* Piwi2	AJW77406.1
Lingula anatina Piwi1	XP_013405373.1	*Drosophila melanogaster* Ago3	NP_001036628.2

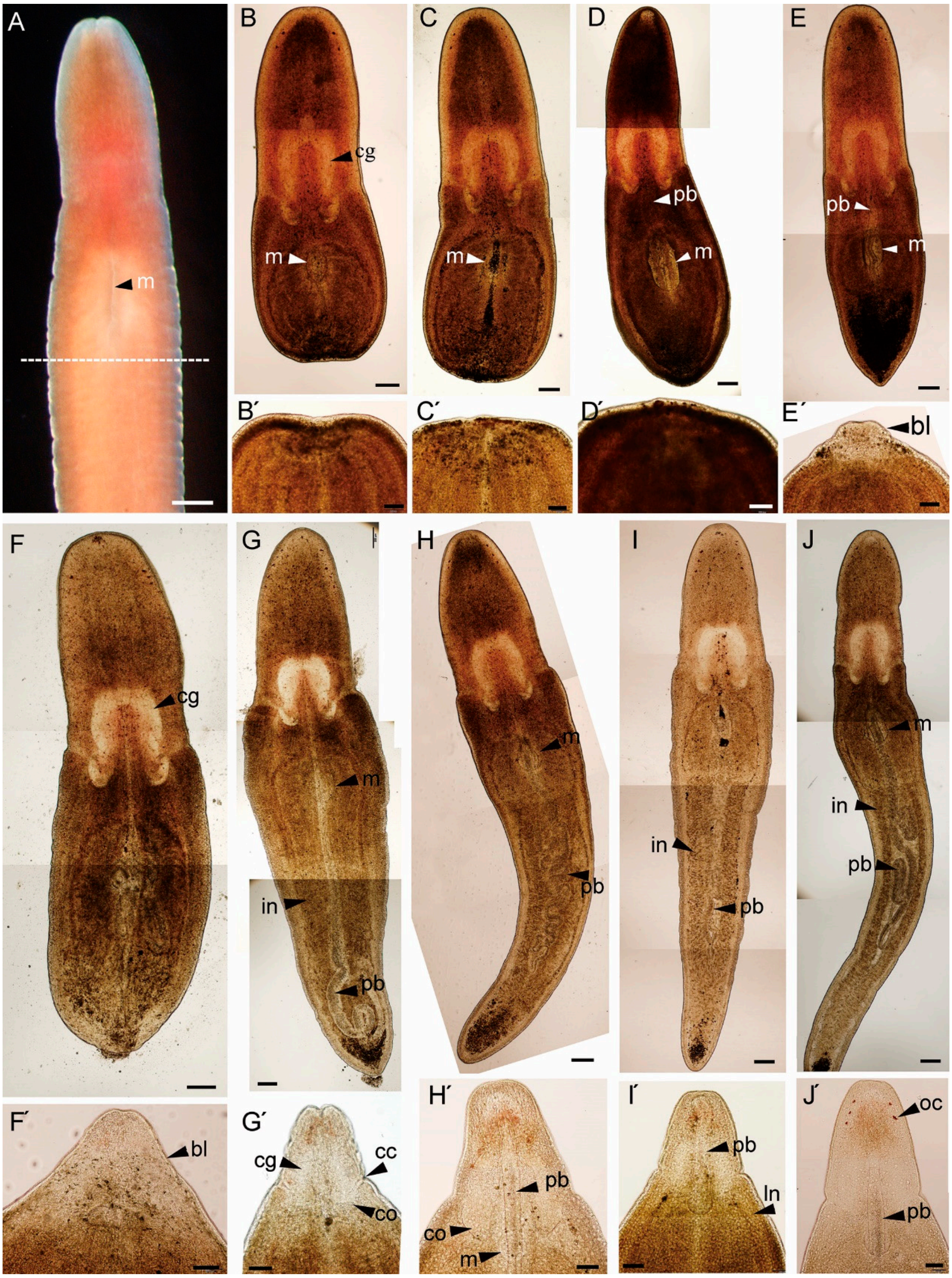

Figure S1. Time course of the regeneration of *Lineus sanguineus* (ventral view). (A) Anterior region of an intact worm showing the cut site (dashed line) of amputation. (B, C, …, J) Posterior regeneration of anterior fragment. (B , C , …, J) Anterior regeneration of posterior fragment. (B and B) 2 d. (C and C) 4 d. (D and D) 6 d. (E and E) 10 d, with obvious blastema. (F and F) 14 d. (G and G) 20 d, posterior regeneration with distinguishable stomach and intestine (G), anterior regeneration with recognizable cerebral ganglia, cerebral organs, rhynchocoel and proboscis (G). (H and H) 26 d. (I and I) 32 d. (J and J) 40 d. Abbreviations: bl, blastema; cc, opening of cerebral canal; cg, cerebral ganglion; co, cerebral organ, in, intestine; m, mouth; pb, proboscis; oc, ocellus. Scale bars: A-J = 200 μm; B -J = 100 μm.

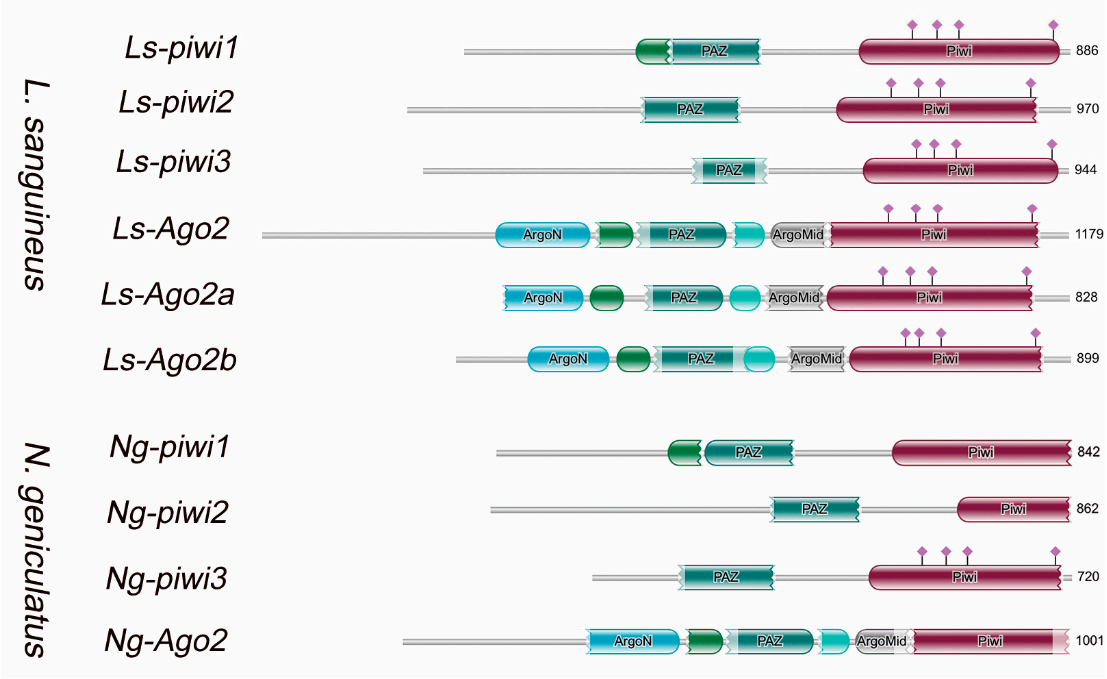

Figure S2. Domain structures of the inferred Argonaute proteins in *Lineus sanguineus* and *Notospermus geniculatus*. The length (number of amino acid residues) of each inferred protein is indicated by the number on the right.

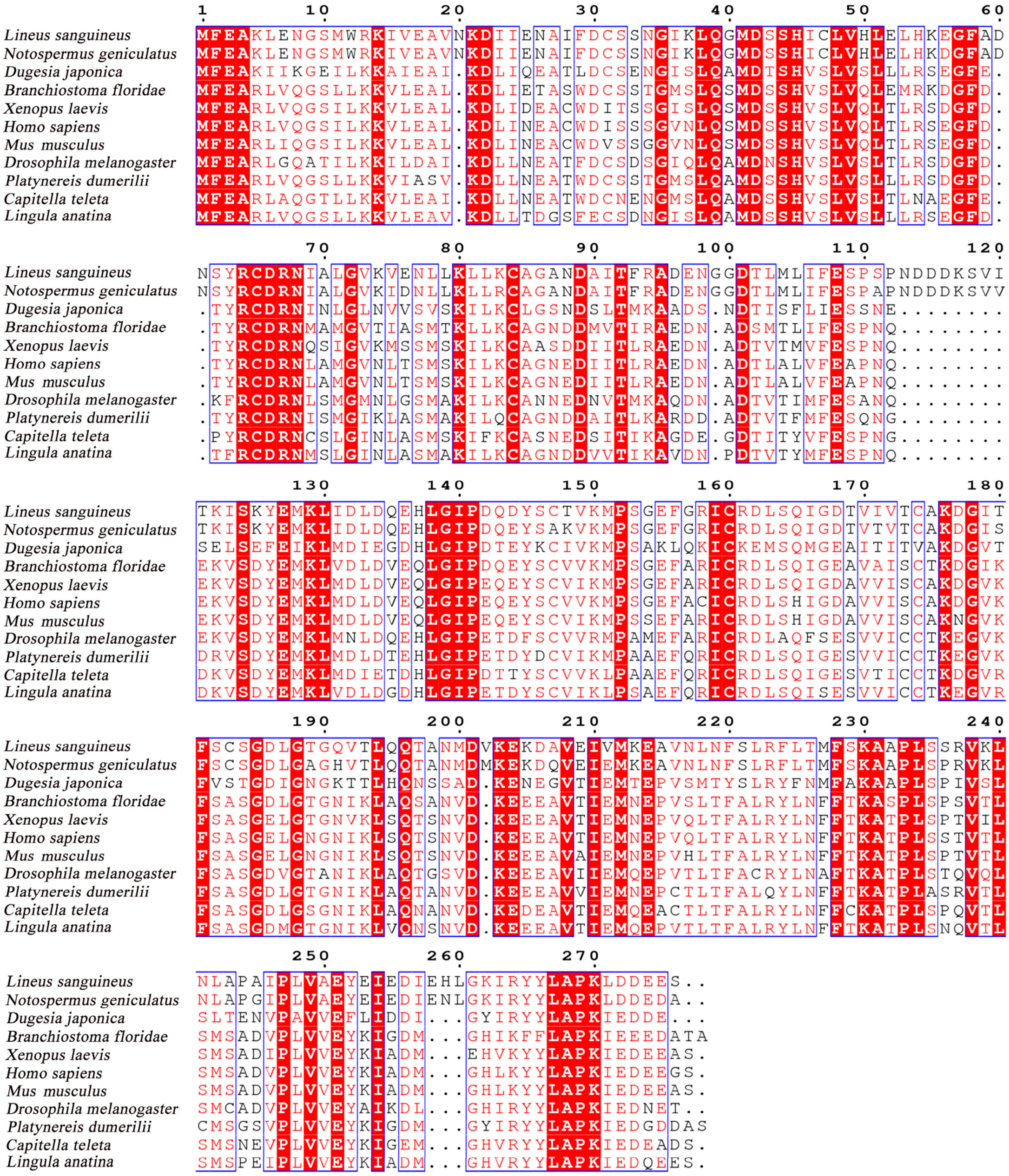

Figure S3. A comparison of the deduced amino-acid sequences of the proliferating cell nuclear antigen (PCNA) of *Lineus sanguineus* and *Notospermus geniculatus* with PCNAs of nine other animals including *Dugesia japonica* (Accession # BAD89370), *Branchiostoma floridae* (Accession # XP_002588880), *Xenopus laevis* (Accession # P18248.1), *Homo sapiens* (Accession # CAG38740), *Mus musculus* (Accession # EDL41327), *Drosophila melanogaster* (Accession # NP_476905), *Platynereis dumerilii* (Accession # CCV20094), *Capitella teleta* (Accession # ELT99750), *Lingula anatina* (Accession # XP_013397320).

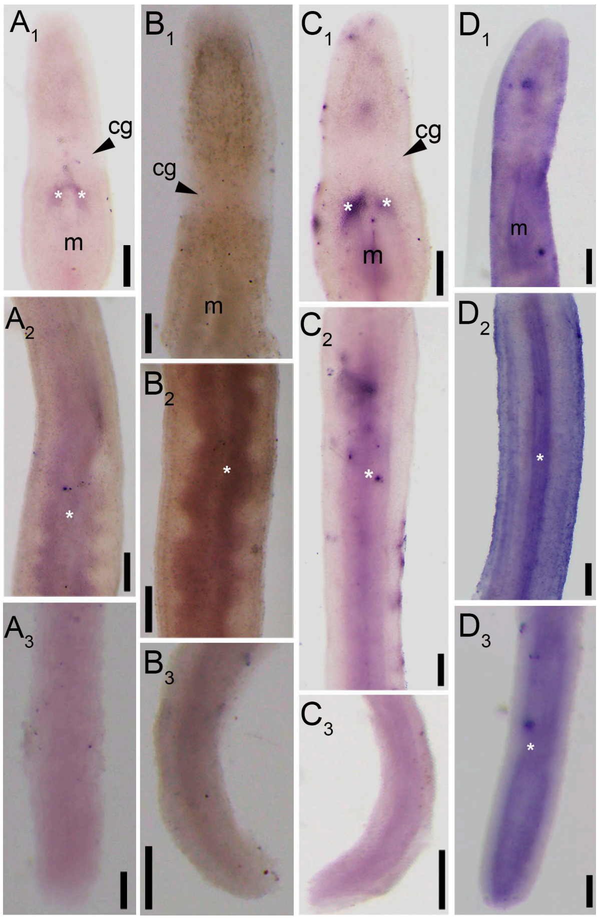

Figure S4. Micrographs for sense-probe staining (control) of four genes in *Lineus sanguineus*. (A) *Ls-pcna*, with unspecific binding in blood lacuna (A1), diffuse staining in intestine (A2). (B) *Ls-piwi1*, with diffuse staining in intestine (B2). (C) *Ls-piwi2*, with unspecific binding in blood lacuna (C1), diffuse staining in intestine (C2, C3), and unexpected spots on body surface (C1, C2). (D) *Ls-piwi3*, with unspecific binding in rhynchocoel (D2) and diffuse staining in intestine (D3). Mentioned staining marked with asterisks. Abbreviations: cg, cerebral ganglia; m, mouth. Scale bars = 200 μm.

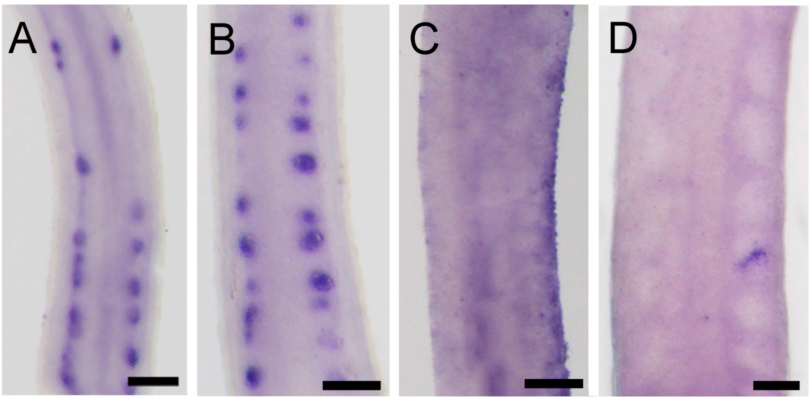

Figure S5. Expression patterns of *Ls-pcna*, *Ls-piwi1*, *Ls-piwi2*, and *Ls-piwi3* in the intestinal region of non-starved *Lineus sanguineus*. (A) *Ls-pcna*. (B) *Ls-piwi1*. (C) *Ls-piwi2*. (D) *Ls-piwi3*. Scale bars: A-C = 200 μm; D=100 μm.
